# Endometriosis-associated Ovarian Clear Cell Carcinoma: A Special Entity?

**DOI:** 10.7150/jca.61107

**Published:** 2021-09-23

**Authors:** Yue Sun, Guoyan Liu

**Affiliations:** 1 Department of Gynecology and Obstetrics, Tianjin Medical University General Hospital, Tianjin, 300052, China.; 2 Tianjin Key Laboratory of Female Reproductive Health and Eugenics, Tianjin, 300052, China.

**Keywords:** endometriosis, ovarian clear cell carcinoma, malignant transformation, clinical features

## Abstract

Endometriosis is an estrogen-dependent disease, which serves as a precursor of ovarian cancer, especially clear cell carcinoma (OCCC) and endometrial carcinoma. Although micro-environmental factors such as oxidative stress, immune cell dysfunction, inflammation, steroid hormones, and stem cells required for malignant transformation have been found in endometriosis, the exact carcinogenic mechanism remains unclear. Recent research suggest that many putative driver genes and aberrant pathways including ARID1A mutations, PIK3CA mutations, MET activation, HNF-1β activation, and miRNAs dysfunction, play crucial roles in the malignant transformation of endometriosis to OCCC. The clinical features of OCCC are different from other histological types. Patients usually present with a large, unilateral pelvic mass, and occasionally have thromboembolic vascular complications. OCCC patients are easier to be resistant to chemotherapy, have a worse prognosis, and are usually difficult to treat. To improve the survival of OCCC patients, it is necessary to better understand its specific carcinogenic mechanism and explore new treatment strategy, including molecular target.

## Introduction

Endometriosis is a common estrogen-dependent disease in which endometrium grows outside the uterus. This complex disease affects approximately 7-15% of women of reproductive age. Although endometriosis is identified as a morphologically benign disease, it has characteristics similar to invasive neoplasms, such as peritoneal implants, local invasion and distant metastasis. Evidence suggests that endometriosis, especially ovarian endometriotic cyst, may be a monoclonal neoplastic disease with premalignant potential 1. Therefore, the risk of development of ovarian cancer arise from endometriosis cannot be ignored.

A large-scale epidemiological study using more than 20,000 women with endometriosis first found that the incidence of ovarian cancer has increased significantly 2. Subsequent studies have found that 5-10% of endometriosis patients have ovarian cancer, but the number is sometimes different. Studies have shown that endometriosis increases the risk of ovarian cancer by about 1.2-1.8 times 3. During follow‑up of endometrial cysts in Murakami's study, 75% of patients progressed to ovarian cancer within 5 years and most patients progressed within 10 years 4.

Endometriosis is a risk factor for ovarian cancer. It is believed that endometriosis-associated ovarian cancer (EAOC), most commonly ovarian clear cell carcinoma (OCCC), develops from ovarian endometrial cysts. OCCC is a specific pathological type of epithelial ovarian carcinoma (EOC) with unique clinical and molecular features 5. Patients usually present with a large, unilateral pelvic mass, and occasionally have thromboembolic vascular complications or hypercalcemia 6, 7. OCCC displays chemo-resistance to platinum, the efficacy of platinum-based chemotherapy is only 20% to 50% for OCCC. OCCC patients have a worse prognosis, and are usually difficult to treat 8, 9. The molecular changes in OCCC have not been completely elucidated. A deeper understanding of pathology and mechanisms of carcinogenesis is needed to develop a specific treatment strategy, including molecular targeting. Considering the increasing attention to this topic, the article summarizes the current knowledge about the problem.

## Epidemiology of endometriosis-associated OCCC

OCCC is the second most common EOC, first recognized as a unique histologic subtype by the World Health Organization in 1973 10, 11. The prevalence of OCCC differed by region. It accounts for 3.1%-11.1% of EOC in the United States, but it has a higher prevalence in East Asia, accounting for 25%~30% and 10.3%~11.6% of EOCs in Japan and Korea, respectively 12-14. A possible link between endometriosis and OCCC in certain cases has been suggested for a long time. Since Sampson described an EAOC for the first time in 1925, multiple studies have assessed the incidence of endometriosis-associated OCCC 15. In a large register study of 20,686 Swedish women with endometriosis, the standardized incidence ratio for developing ovarian cancer during a mean follow-up of 11.4 years was 1.9 (95% CI: 1.3-2.8) 16. A pooled meta-analysis of 13 case-control studies including 7,911 women with ovarian cancer and 13,226 controls demonstrated that self-reported endometriosis was associated with a significantly increased risk of OCCC (odds ratio 3.05, 95% CI 2.43-3.84, p<0.0001) 17. Consistent observations were reported by a Danish register study, confirming that endometriosis was associated with increased risks for ovarian cancer (OR 1.34; 95% CI: 1.16-1.55), due primarily to clear-cell types (OR 3.64; 95% CI: 2.36-5.38) 18. In the ENOCA population-based cohort study, the incidence of OCCC in a cohort of 131,450 women with a histological diagnosis of endometriosis was compared to an age-matched control cohort of 132,654 women with a benign dermal nevus. The age-adjusted incidence rate ratio (IRR) was 7.18 (95% CI: 6.17-8.46) for ovarian cancer in women with endometriosis, and OCCC had the higher age-adjusted incidence rate ratio of 21.34 (95% CI: 14.01-32.51) 19. In summary, the lifetime risk for developing ovarian cancer is low with approximately 1.9%. However, the risk for a woman with endometriosis to develop ovarian cancer is up to 50% higher than in the general population. This is particularly true regarding the risk for developing OCCC, where the risk is tripled, respectively.

## Pathological evidence that OCCC arises from endometriosis

In 1925, the criteria for cancer arising from endometriosis was first proposed by Sampson, including that: (1) the tumor is adjacent to unequivocal foci of endometriosis, (2) there is no other primary tumor, (3) there are tissues resembling endometrial stroma surrounding epithelial glands 15. Scott revised the criteria more stringently, requiring the transition from endometriosis to neoplastic epithelium 20. Since LaGrenade reported the pathological continuation of atypical endometriosis to cancer lesions in seven cases of EAOC, more and more evidence supports that atypical endometriosis is an intermediate lesion between endometriosis and ovarian cancer 21. First, compared with benign endometriotic cysts, atypical endometriosis is more common in endometriosis accompanied by malignant tumors 22, 23. Atypical endometriosis was seen in 61% of EAOC, while it was observed in only 1.7% of endometriosis cases without cancer 23. Substantial pathological data have showed the continuous transition from benign endometriosis to cancer, and atypical endometriosis may be observed in these areas 24. Among the cancers, OCCC and endometrioid carcinomas are predominant for unknown reasons. Molecular analysis also indicated that there were multiple genetic events in atypical endometriosis, suggesting that the lesion has intermediate properties between benign endometriosis and ovarian cancer. Similar somatic mutations were identified in OCCC and adjacent atypical endometriosis, demonstrating that OCCC is not derived from the ovarian epithelium; rather, it represents tumor cells transformed from displaced endometriotic tissues 11, 25. Therefore, atypical endometriosis is a precancerous lesion of OCCC.

## Pathogenesis involved in the malignant transformation of endometriosis to OCCC

**Figure 1:**
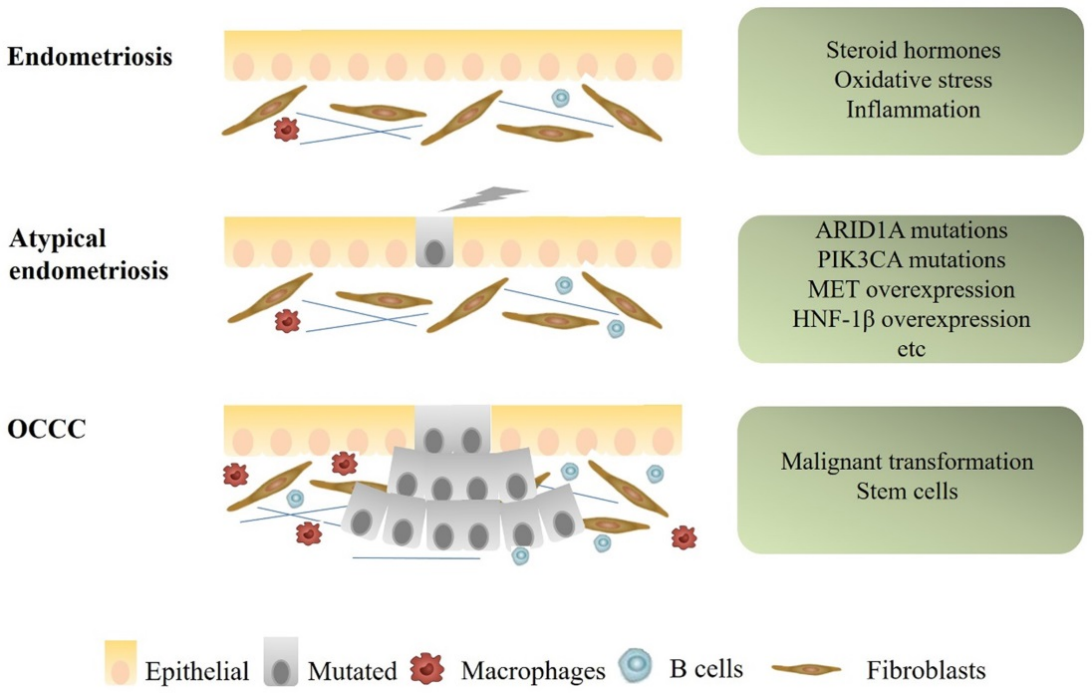
** Mechanisms of progression from endometriosis to endometriosis-associated OCCC.** Some microenvironmental changes, as well as several genetic alterations, such as ARID1A, PIK3CA mutations, and MET, HNF1β overexpression, were suspected to be associated with early carcinogenic events of OCCC.

There are currently two main mechanisms to explain the relationship between OCCC and endometriosis. One is that endometriosis and OCCC share many of the same risk factors, including early menarche, late menopause and infertility. The common risk factors result in the occurrence of both diseases. Use of oral contraceptives, tubal ligation, and hysterectomy could decrease the risk of both diseases 26. The other is that endometriotic cells gradually transform into cancer cells. The process of transformation from typical endometriosis, through atypical endometriosis, finally to OCCC seems to be mainly related to specific micro-environmental factors, molecular alterations and presence of stem cells. This article will focus on this part (Figure 1).

## Alterations of microenvironment events

**Figure 2:**
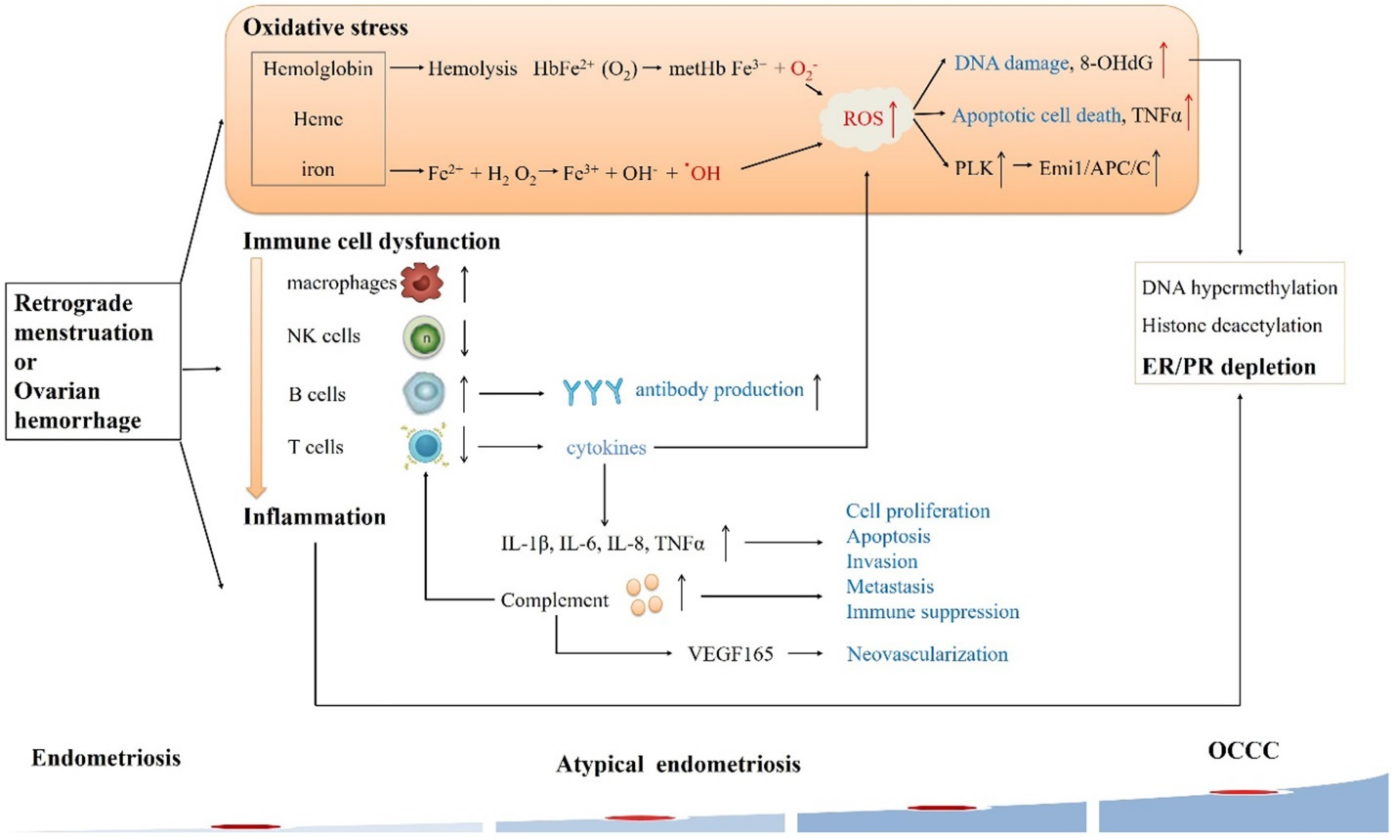
** Alterations of microenvironment events in endometriosis-associated OCCC.** In the endometriotic cyst, endometrial cells are exposed to the stresses of hypoxia, immune cell dysfunction and inflammation, eventually leading to OCCC.

The most well-known implantation theory about malignant transformation of endometriosis to OCCC has now been widely accepted, which posits that the viable menstrual endometrial cells were deposited in the pelvic cavity via retrograde menstruation and became the origin of ectopic endometrial tissue. These shed menstrual endometrial cells still capable to attach to the peritoneum, invade, proliferate, and differentiate 27. The ovary is probably favored seeding sites for endometriosis cells especially in the ovulation sites 28. Repeated bleeding of endometriosis cysts during the menstrual cycle causes changes in the microenvironment, resulting in the malignant transformation of endometriosis to OCCC (Figure 2).

### Oxidative stress

In the reproductive period, repeated hemorrhaging in endometriotic cysts induces excessive iron, cell-free hemoglobin, and heme accumulation. They are prone to autoxidation and might spontaneously convert oxyHb to metHb. Autoxidation of hemoglobin continuously generate ROS (O_2_^-^). Iron derivatives also stimulate Fenton reaction, increasing ROS (˙OH) production in endometriotic cyst. ROS could react with proteins, lipids and especially DNA, forming a possible source of carcinogenic mutations in the genome^29^. An oxidative stress marker as well as a biomarker of carcinogenesis, 8-hydroxydeoxyguanosine (8-OHdG) is formed when ROS interacts with DNA. Yamaguchi evaluated the contents of endometriotic cysts and found that free iron, 8-OHdG, lipid peroxidase, and lactose dehydrogenase levels were elevated compared to nonendometriotic cysts^30^. In addition, ROS can rapidly activate Polo-like kinases (PLK, a mitotic regulator) by regulating DNA replication under stressful conditions, thereby promoting genome stability. PLK phosphorylates Emi1 (Early mitotic inhibitor-1) to ensure that S-phase and mitosis entry are promoted by suppressing the anaphase-promoting complex/cyclosome (APC/C). Overexpression of Emi1 leads to mitotic catastrophe and genome instability and promotes tumorigenesis 31. The latest data suggest that the Emi1/APC/C pathway is upregulated in atypical endometriosis during OCCC tumorigenesis 32. Moreover, oxidative stress appears to be able to decrease the expression of ARID1A protein and mRNA levels in endometriotic cells, and low ARID1A gene activity in endometriosis may be a predisposing factor for the increased susceptibility of these lesions to the malignant transformation 33.

Hemoglobin and heme also alter the expression of many genes. Using microarray analysis, Mandai discovered 437 genes that are differentially expressed in OCCC. These genes were mainly related to oxidative stress and inflammation, which indicated that the cancer specifically expresses stress responsive genes 1. Kajihara further supported the result that 87% of highly upregulated genes found in the OCCC are redox related genes, including oxidase and detoxification enzymes 34. Accordingly, we speculate that the content of endometriotic cysts creates an environment of high oxidative stress, exposing epithelial cells to constant oxidative stress that may trigger the malignant transformation. OCCC acquires the ability to resist stress during the process of carcinogenesis in a stressful microenvironment, which may explain the observed OCCC chemotherapy resistance. However, the hypothesis should be further verified later.

### Immune cell dysfunction and inflammation

Endometriosis is related to activated immune cells and abnormal cytokines in the peritoneal fluid, thereby forming a local inflammatory environment 35. Inflammation of coelomic epithelial cell-derivatives in the female reproductive tract is a major contributor to malignant transformation in endometriosis-associated OCCC 36. There is substantial evidence of immune cell dysfunction in women with endometriosis: reduced T cell reactivity and NK cytotoxicity, increased B cell polyclonal activation and antibody production, increased number and activation of peritoneal macrophages, and changes in apoptotic pathways, which contribute to OCCC development 35, 37-41. Endometrial fragments released during endometriosis can cause inflammation in the peritoneal cavity via neutrophils and macrophages recruited to the area 42. However, peritoneal macrophages and NK cells in endometriosis cannot completely eliminate endometrial cells or fragments. The imbalance of T cells may result in abnormal secretion of cytokines (TNF-α, IL-8 and VEGF, etc.) and inflammation, leading to endometrial lesions 43. In addition, increased levels of several important cytokines such as IL-1β, IL-2, IL-6, IL-8, and TNF-α in acute inflammatory phase have been found in peritoneal fluid of patients with endometriosis 44. Similar to results in endometriosis, numerous studies suggest inflammatory cytokines promote the growth and progression of epithelial ovarian cancer, implying that inflammation is involved in the development of this disease 45. Moreover, inflammatory cells may promote cell proliferation, angiogenesis, invasion, metastasis, production of ROS and inhibit apoptosis, contributing to DNA damage and mutations.

In recent years, studies have revealed new features of inflammation and pointed out previously unknown roles of complement in endometriosis and EAOC 46, 47. Complement proteins are abundant in epithelial cells of both benign and malignant lesions. Suryawanshi performed the immune gene expression analysis about pelvic inflammation firstly, and found five out of the total of nine genes differentially expressed in endometriosis and EAOC are complement genes, supporting the importance of complement cascade in these diseases. They further revealed that chronic inflammation in endometriosis is mainly determined by complement. Complement is still active in EAOC, while tumors with serous histology are not active, which further prove the heterogeneity of EAOC 47. The complement system may contribute to the development of cancer through multiple mechanisms coexisting in the tumor environment either directly, by promoting tumor cell proliferation, or indirectly, by stimulating immune suppression and neovascularization 48, 49. The production of complement C5a in the tumor microenvironment enhances tumor growth through inhibiting anti-tumor CD8+ T cell mediated response, and pharmacological blockade of C5a receptor significantly delays tumor growth 50. In addition, complement inhibition blocks tumor growth by altering vascular endothelial cell function and VEGF165 expression 50. And Su's recent study suggested that complement-activation-alternative-pathway may be the crucial dysfunctional immunological pathway in duality for carcinogenesis at all OCCC stages 51.

Taken together, immune factors are obviously involved in the pathogenesis of endometriosis and OCCC. Many promising immune biomarkers may serve as potential therapeutic targets for the transition of endometriosis to OCCC in the future.

### Steroid hormones

Endometriosis is an estrogen-dependent disease, and estrogen is closely related to the pathogenesis of ovarian cancer. However, estrogen receptors (ER) is significantly downregulated in OCCC compared to normal ovaries, endometriosis, therefore, endometriosis could become hormone-independent during the malignant transformation process 52. Lack of hormone functioning may be a turning point in the development of OCCC 53. The high expression of ER was retained in both distant and adjacent endometriotic lesions, but was lost in the primary OCCC, indicating a late carcinogenic event. The hypothesis concerning the carcinogenic pathogenesis of OCCC is that heme- and iron-mediated oxidative stress and sustained inflammatory reaction oxidatively modify DNA, lipids, and proteins, resulting in DNA hypermethylation, histone deacetylation or ER depletion 54. Low ER expression may explain the reason for the poor prognosis of OCCC.

## Alterations of molecular events

**Figure 3:**
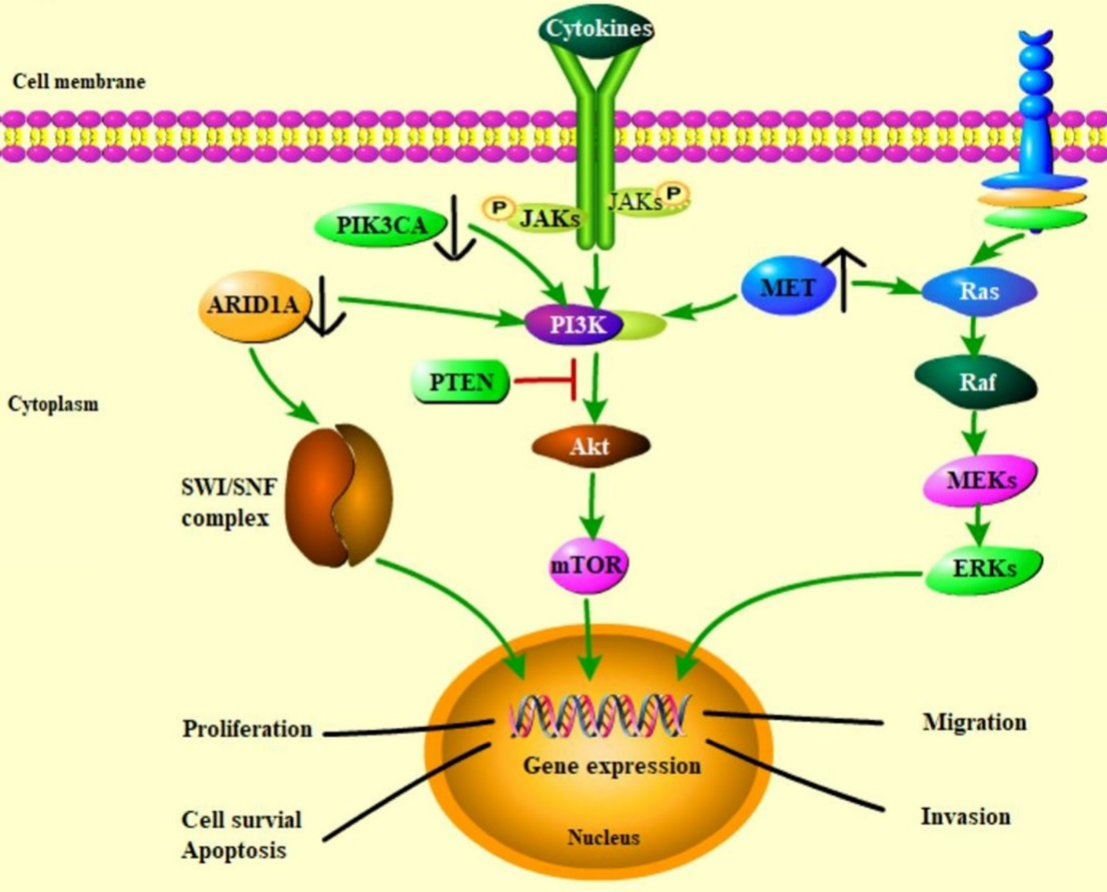
** Alterations of several molecular events in endometriosis-associated OCCC.** Genomic events frequently occurring in OCCC, such as ARID1A, PIK3CA mutations, and MET overexpression, are possibly regulated by interacting pathways.

**Figure 4:**
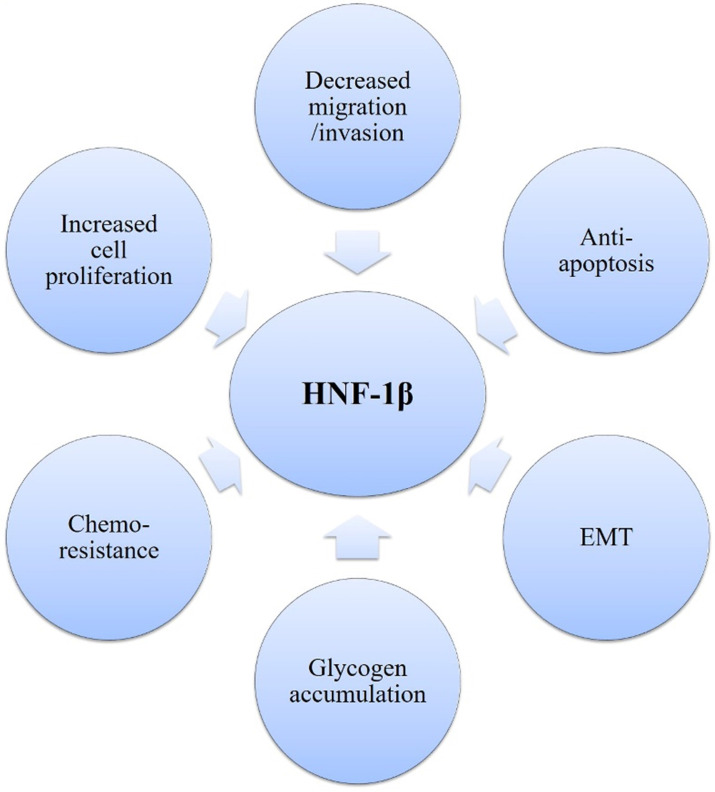
Pleiotropic role of HNF1β in establishing the OCCC phenotype.

Some somatic mutations have been detected in paired eutopic and ectopic endometrium, and ectopic tissue has a higher mutation burden 55. Endometriotic lesions commonly carry multiple somatic mutations; atypical endometriosis and co-existing tumors share nearly all of the somatic mutations, such as driver mutations in ARID1A, PIK3CA and high expression of MET and HNF1β, and it is thought that those above mutations occurred early in the malignant transformation of the OCCC (Figure 3 & 4, Table 1) .

### ARID1A

ARID1A (also known as BAF250a) is a ubiquitously expressed 250 kDa protein that functions as part of the mammalian SWItch/Sucrose Fonfermentable Complex (SWI/SNF). This complex plays a major role in DNA repair either by promoting the accessibility of DNA on the chromatin directly or by improving the functions of DNA repair proteins, such as P53, GADD45, BRCA1 and Fanconi Anemia proteins indirectly 56. By changing the accessibility of chromatin, it also regulates many cellular processes such as proliferation, differentiation, development and tumor suppression 56-58. In 2010, ARID1A was first reported to be frequently mutated in OCCC. ARID1A mutations were found in 55 of 119 (46%) OCCC using whole transcriptome sequencing in a report from the Canadian Ovarian Cancer Research tumor bank 25. Simultaneously, a second report showed that ARID1A mutations could be detected in 24 of 42 (57%) OCCC 59. Additionally, these authors were able to show that, in two cases of OCCC with available adjacent atypical as well as distant endometriosis, identical ARID1A mutations were found in the cancer and the adjacent endometriosis but were absent in the distant endometriotic lesions, implying that ARID1A mutations play a role in the malignant transformation of endometriosis. A study including 54 patients demonstrated a progressive increase in ARID1A protein expression loss from normal endometrium (0%), to typical endometriosis (19%), to atypical endometriosis (38%), further supporting that ARID1A loss is an early event in OCCC pathogenesis 60. ARID1A mutations or loss of protein expression have been reported in a wide range of gynecological and other malignancies over the past 3 years, firmly establishing ARID1A as a frequently mutated tumor-suppressor gene 61, 62.

Several studies have shown that ARID1A mutations are frequently in tumors that exhibit microsatellite instability (MSI) and often coexist with PIK3CA mutations. In Jones's study, 14 of 24 (58%) tumors with ARID1A mutations also carried PIK3CA mutations, while 18 (17%) had no mutations 59. In agreement with previous results, 71% of tumors without ARID1A expression were found to have PIK3CA mutations compared to 44% of those with intact ARID1A expression in 42 OCCC patients 63. The same pattern was also observed in uterine cancers with ARID1A mutations, which were enriched in a series of 222 cases of uterine cancer with PIK3CA and PTEN mutations 64. In this series, ARID1A mutations were associated with phosphorylation of multiple members of phosphatidylinositol 3-kinase (PI3K), which means that ARID1A activates the PI3K pathway independent of PTEN and PIK3CA aberrations 64. This is corroborated by subsequent studies which showed that loss of ARID1A expression usually coexisted with PI3K-AKT pathway activation 65. AKT phosphorylation was increased in OCCC with ARID1A loss that was independent of the PTEN and PIK3CA status 66. In addition, AKT phosphorylation in response to ARID1A knockdown has recently been reported in breast cancer and lung cancer cell lines 67, 68.

The functional consequences of ARID1A loss in OCCC are unclear. Consistent with its proposed tumor suppressor effect, the re-expression of ARID1A in the OCCC cell line OVISE with ARID1A mutations resulted in growth inhibition 69. Similarly, knockdown of ARID1A in the two IOSE cell lines led to increased proliferation *in vitro* and increased tumorigenicity when implanted subcutaneously in nude mice 69. Recently reports has found that ARID1A knockdown promotes proliferation and regulates the cell cycle, especially G2/M checkpoint-related genes, such as AURKA, PLK1, PLK4, CCND1 and CCNB1 in mouse endometrium and human IOSE cell lines 70.

In summary, ARID1A loss appears to be a driving event in approximately half of OCCC cases. Clinically, there does not appear to be a distinct ARID1A-driven OCCC phenotype yet. Current research focuses on associating the ARID1A mutation with other mutations in OCCC and using them as prognostic factors for malignant tumors. In a study of OCCC, ARID1A mutations are related to platinum-based chemo-resistance, and these patients tend to have a shorter survival 71. Although ARID1A mutation has not been considered as a prognostic factor for ovarian cancer, studies on breast cancer have shown that the lack of BAF250a is related to the low survival rate of postoperative disease, suggesting that it may be a breast cancer treatment target 72. Once we have clearly demonstrated the mechanisms of malignant transformation caused by ARID1A mutations in OCCC, we can explore them to develop novel targeted therapies.

### PIK3CA

Mutations in ARID1A are considered as an early event but inactivation of ARID1A alone is not enough to initiate the oncogenic transformation of either the endometrium or ovarian surface epithelium. Other mechanisms such as PIK3CA-activating mutations in cooperation with loss of ARID1A expression seem to be necessary to promoting the occurrence of OCCC 36. Frequent PIK3CA mutations in OCCC were first reported in 2009 and further studie have confirmed an incidence of PIK3CA mutations in OCCC between 33 and 43% 73, 74. The same mutations are also detected in the atypical endometriosis adjacent to OCCC but not in endometriosis distant from OCCC or solitary endometriosis, implying that they occur early in OCCC pathogenesis 75.

PIK3CA encodes a catalytic subunit of PI3K, mutations of which lead to increased kinase activity, in turn stimulating downstream AKT and increasing cell invasion and metastasis. Therefore, it plays a vital role in the occurrence and development of tumors, and are of great significance for clinical diagnosis, treatment and prognosis. Several studies have observed the activation of the PI3K/AKT pathway in endometriosis and OCCC 76. PIK3CA mutations often occur simultaneously with ARID1A mutations, resulting in the PI3K/AKT pathway activation in OCCC 73, 77. The PI3K/AKT pathway is the main regulator in cancer cell growth, proliferation, differentiation, motility, survival and glucose metabolism 78. PI3K could regulate the progression of G1 cell cycle and the expression of cyclin by activating the AKT/mTOR /p70S6K1 signaling pathway in ovarian cancer cells. It could also promote cell survival via several ways, including phosphorylation and inactivation of the proapoptotic proteins Bad and caspase-9 78, 79. In addition, loss of ARID1A-encoded protein could influence pAKT activation, γH2AX alteration, and concomitant apoptosis pathway activation 80. Moreover, activation of this pathway is strongly associated with a reduction in chemotherapy response in ovarian cancer 81. With regards to outcomes, two series of 56 and 62 OCCC cases showed improved prognosis in OCCC patients with PIK3CA mutations 82, 83. However, no differential activity of the PIK3CA inhibitors LY294002 and BEZ235 or the mTOR inhibitor temsirolimus was observed in a panel of OCCC cell lines with or without PIK3CA mutations 83. Further research is needed to provide preliminary clinical evidence of benefit from targeting PIK3CA.

### MET

As a proto-oncogene, MET encodes a receptor tyrosine kinase c-MET for hepatocyte growth factor (HGF), which appears to be the most frequently amplified gene in OCCC. MET overexpression and gene amplification are usually observed at a frequency of 22% and 24%-37% in OCCC, respectively 84, 85. Furthermore, in cases of endometriosis and adjacent OCCC components, the incidence of MET overexpression is gradually increasing. MET overexpression are observed at a frequency of 0% in the precursors of nonatypical form, 67% in atypical form, 92% in the relatively differentiated carcinoma components and 100% in the poorly differentiated carcinoma components, supporting the notion that MET is one of the key drivers of carcinogenesis 86. When HGF binds with overexpressed MET, the Raf/Ras/mitogen-activated protein kinases (MAPK) pathway, the PI3K/AKT/mTOR signaling pathway and several other pathways are activated, thus stimulating the proliferation, migration, and invasion of cancer cells 87. These results indicate that MET alterations may promote the development of the MET amplification-positive OCCC 86. In addition, it has been reported that both MET overexpression and gene amplification are associated with adverse outcomes for OCCC patients 88, 89. The survival rate of stage I and II OCCC patients with MET gene amplification is significantly lower compared to that of patients without MET gene amplification 84. Knocking out MET expression by shRNA can increase cellular apoptosis and senescence and thus reduce the viability of OCCC cells 90. Yamashita also observed AKT2 gene amplification in 5 of 21 samples (13 OCCC primary tumors and 8 OCCC cell lines) through array-based comparative genomic hybridization analysis 90, suggesting that MET-amplified OCCC cells mainly depend on the MET/PI3K/AKT pathway activation to regulate cell proliferation and survival. Targeted drugs that inhibit the MET/PI3K/AKT pathway are expected to be used in the clinical treatment of OCCC.

### HNF-1β

HNF-1β is a transcription factor, which is involved in embryonic development and tissue-specific gene expression in multiple organs, including the ovaries. In 2003, it was first reported that nuclear IHC staining for HNF-1β was positive in 20 of 21 OCCC cases compared with only 1 of 61 non-OCCC EOC 88. Subsequent reports have confirmed that over 95% of OCCC stain positive for HNF-1β, whereas other EOC subtypes do so extremely rarely 91. Furthermore, in a series of 12 OCCC cases with available adjacent endometriosis, nine showed positive staining for HNF-1β in the nonmalignant endometriotic epithelium, and 16 of 40 benign endometriotic cysts also showed HNF-1β positivity 91. Expression of HNF-1β was identified in endometriosis, atypical endometriosis and OCCC, implying a shared molecular pathogenesis. Early differentiation to the clear cell lineage primarily occurs in ovarian endometriosis, which may explain why OCCC frequently occurs in ovarian endometriosis 91.

As OCCC is characterized by HNF-1β overexpression, HNF-1β is a useful molecular marker that could help diagnose OCCC 91, 92. The hypomethylation of HNF-1β CpG island participates in upregulation of HNF-1β in OCCC 93. Previous studies have demonstrated that HNF-1β regulates the gene/protein expression such as annexin A4, osteopontin, uridine diphosphate (UDP)-glucuronosyl transferase 1A1 (UGT1A1), and insulin-like growth factor-binding protein 1 (IGFBP-1), which are vital for cancer progression 88. Many biological functions of OCCC also depend on HNF-1β and are possibly regulated by interacting pathways (Figure 4). HNF-1β is critical for cell survival in endometriosis and OCCC and involves the features of OCCC, including glycogen accumulation in cancer cells, strong anti-apoptotic activity and chemo-resistance due to lower cell proliferation 88. First, HNF-1β could promote glucose uptake and glycolysis, contributing to cell survival under hypoxic conditions or stressful environments. Moreover, HNF-1β overexpression leads to detoxification to overcome persistent inflammation and oxidative stress, which are required for carcinogenesis of endometriosis. HNF-1β also plays an essential role in the anti-apoptosis of OCCC. Reduction of HNF-1β mRNA induced apoptosis of OCCC cells 88. Upregulation of HNF-1β by NF-κB regulates the susceptibility to apoptosis via altering bcl-2 expression in OCCC 94.

Moreover, HNF-1β appears to be one of the main drivers of gene expression programs in OCCC. The gene expression profiling study reported a significant overlap between their 25-gene HNF-1β signature, derived from overexpression in kidney HEK293 cells, and previously reported OCCC expression profiles 88, 95. Gene expression signatures derived from HNF-1β knockdown in EOC RMG-I and RMG-II cell lines could distinguish OCCC cases from other EOCs in two publicly available ovarian cancer datasets 96. In addition, HNF-1β was shown to drive gene expression profiles similar to the epithelial-mesenchymal transition (EMT) in SKOV3 cells, leading the authors to conclude that HNF-1β is important for maintaining the E-cadherin-expressing epithelial phenotype in OCCC 97.

In summary, HNF-1β overexpression is a defining characteristic of OCCC, driving gene-expression signatures and having a major impact on OCCC proliferation, apoptosis and metabolic profiles. However, no specific inhibitors of HNF-1β have yet been developed, and the potential of HNF-1β as a new therapeutic target must be supported by further studies.

### MiRNAs

It has been discovered that new regulators of gene expression, miRNAs, may be involved in the transformation of endometriosis into OCCC 98, 99. MiRNAs have been implicated to play an important role in different pathological and physiological processes, such as cell proliferation, apoptosis, hypoxia, inflammation, angiogenesis, extracellular matrix remodeling, and tissue repair, and their dysregulation points to various pathologies, including endometriosis and EAOC. However, which miRNA is actually related to EAOC remains controversial. MiR-21 and miR-214 are significantly upregulated in EAOC and can suppress the expression of PTEN 100. As a well-known tumor suppressor gene, PTEN inactivation is an early event in the malignant transformation of endometriosis 101. Another miRNA significantly upregulated in endometriosis and EAOC patients compared with healthy controls is miR-191 98. Dong found that miR-191 could directly regulate TIMP3 expression in both endometriosis and EAOC cells 102. TIMP3 is a member of the metalloproteinase family and may be a key factor regulating cell proliferation and invasion of endometriosis and EAOC cells. Through modulating TIMP3, miR-191 could also promote cell proliferation and invasion of endometriosis cells. The miR-191-TIMP3 axis might take an important part in the pathological development of endometriosis to EAOC. Further studies indicated that miR-30a, miR-30c, miR-31, miR-486-5p, miR-532-5p, and miR-885-5p were also increased in OCCC. MiR-30 was five-fold overexpressed in OCCC 103. Sestito showed that miR-30a overexpression delayed tumor formation in xenograft tumors, and made ovarian cancer cells sensitive to chemotherapy 104. The expression of miR-486-5p in serum and ascites samples from EAOC patients was higher compared to that from ovarian endometrioma patients. Upregulation of miR-486-5p significantly increased cell proliferation and migration, which might act as an oncogenic miRNA in EAOC 105. Overexpression of miR-532 inhibited ovarian cancer cell proliferation, invasion and tumor growth, while miR-532 downregulation was correlated with poor survival in ovarian cancer patients 106. Compared to eutopic endometrium, miR-126 was significantly decreased in endometriosis, which induced the proliferation, migration, and invasion of non-ovarian cancer cells through PI3K, KRAS, or VEGF 107. Additionally, although ovarian cancer patients were not included in the research, reduced miR-126 level was an essential predictor of poor survival in cancer patients 108. Thus, miRNAs may be useful tools in detecting OCCC, but further research should be conducted to validate the possibility.

### Others

A recent study compared various molecular changes in endometriosis-associated OCCC through immunohistochemistry. Five novel biomarkers, eEF1A2, PTCH2, PPP1R14B, PPP2R1A, and XRCC5 are strongly overexpressed in OCCC and associated endometriosis but not in benign endometriosis 52. The tissue-specific translation elongation factor eEF1A2 has been viewed as a potential oncogene, overexpression of which is associated with OCCC. The protein elongation network can activate tumorigenesis and inhibit apoptosis 109. Chang also showed that eEF1A2 could interact with peroxiredoxin 1 (Prdx 1) to provide cells with strong resistance to oxidative stress-induced cell death 110. PTCH2 modulates tumorigenesis and is involved in hedgehog signaling, the activation of which is essential for cell proliferation 111. PPP1R14B, as an inhibitor of protein-phosphatase 1, is necessary for cell migration and retraction 112. Mutations in PPP2R1A, a subunit of the serine-threonine phosphatase PP2A that acts as a tumor suppressor by dephosphorylating oncogenes were initially reported to occur in 7% of OCCC 59. A subsequent report confirmed their presence in 4% of OCCC but their functional effects in OCCC are, at present, unknown 113. XRCC5 is a subunit of Ku ATP-dependent DNA helicase heterodimer, used to repair double-stranded DNA breaks. Compared to endometriosis without cancer, XRCC5 was overexpressed in OCCC associated with endometriosis 52. Recent investigations have demonstrated that increased levels of double-strand DNA break repair proteins such as XRCC5 protect ovarian cancer cells from apoptosis induced by genotoxic stress, which may be related to chemo-sensitivity 114. These new proteins may play a role in the pathogenesis of endometriosis-associated OCCC and will be studied in the future.

## Stem cells

Many reports indicate that OCCC is rich in a subset of cancer stem cells, and ovarian cancer stem-like cells could be isolated from OCCC cells 115, 116. None of the current theories about endometriosis clearly explained them. Some recent proposals have suggested that H3K27me3 histone modification involve the process. A large enrichment of H3K27me3 histone-modified gene set was observed in endometriotic cells but it was silent in OCCC 52. H3K27me3 is marker for gene silencing through polycomb group (PcG) complexes 117. These target gene sets were silent in embryonic stem cells and pluripotent cells, but expressed in specific lineages of normal differentiated cells 118-120. A somatic cell reprogramming study has demonstrated that H3K27me3 silencing marker was increased during the cell transition to a pluripotent state 121. The PcG-regulated gene sets need to remain repressed to maintain pluripotency and be ready to be activated during lineage cell differentiation 119. As a mesenchyme maintenance regulator, WT1 was found in these gene sets, which plays a vital role in tissue repair and regeneration 122. Immunohistochemistry confirmed the result that the expression of WT1 was increased in endometriosis compared with OCCC. Therefore, these lineage-specific genes are expressed in endometriosis, while are silenced in OCCC, suggesting that epigenetic reprogramming mechanisms convert differentiated endometriotic cells into pluripotency in OCCC. There are two points worth further study in the process. First, the gene sets regulated by PcG were silenced in ER-negative breast tumors, which was similar to ER-negative OCCC 118. Estrogen signaling may affect the function of PcG. Second, an epigenetic antagonism was observed between polycomb and SWI/SNF complexes in the process of stem cell programming and tumorigenesis 123. ARID1A is exactly a subunit of the SWI/SNF complex and often loses its function in OCCC. However, research in this area does not provide any direct evidence, it is obviously premature to directly target stem cells as a therapeutic tool.

## Clinical features relative to endometriosis-associated OCCC

Endometriosis-associated OCCC seems a special entity whose clinical features are different from other histological types. The currently recognized dual model of Kurman and Shih on the pathogenesis of epithelial ovarian cancer proposes that endometriosis associated ovarian cancer such as low-grade serous, low-grade endometrioid, and clear cell carcinoma usually belong to Type I tumors 124. There are no studies to report the difference between the above three. A comparison of the clinical features of OCCC and ovarian high-grade serous carcinoma is summarized in Table 2. OCCC is more common among Asians. The incidence of OCCC is 3.1%-11.1% in the United States, versus 25%-30% in Japan and 10.3%~11.6% in Korea 12-14. The OCCC patients are often diagnosed at a younger age about 55 years old. Patients with pathologically confirmed endometriosis-associated OCCC are 4 to 8 years younger at diagnosis than those without endometriosis 125. OCCC tends to appear at significantly earlier stages, possibly due to slow tumor growth and frequent presentation of large tumor pelvic masses 126. OCCC has a higher proportion of early-stage tumors compared to serous cancer, and about 60% of OCCC patients have stage I disease 127. The prevalence of vascular thrombotic events in this disease is also increased 127. About 16.4% of Japanese patients have venous thrombosis. The prevalence of vascular thrombotic events is up to 40%, twice as high as epithelial ovarian cancer.

Most patients with endometriosis-associated OCCC have a history of endometriosis. If patients with endometriosis have the following conditions, the possibility of malignancy should be highly considered: (1) early diagnosed or long-standing endometriosis, (2) larger size of pelvic mass (diameter ≥9 cm), (3) a rapid increase of mass in a short period, (4) short-term recurrences after treatment, and (5) postmenopausal status at the time of the diagnosis 128-134. Through a long period follow-up, Varma found an average year for malignant transformation of endometriosis to ovarian cancer was more than 8 years 135. Dyspareunia and dysmenorrhea are more frequently observed in patients with endometriosis-associated OCCC. Women with endometriosis may seek medical help for their symptoms and find that they have ovarian cancer by accident, whereas patients without endometriosis may develop symptoms only at later stages, which explains why most EAOC patients are diagnosed earlier. Moreover, endometriosis-associated OCCC patients are more susceptible to infertility. Brinton found that the incidence of ovarian cancer in infertile women with endometriosis is higher than those without endometriosis 136. Stewart further pointed out that the ovarian cancer risk is reduced among women with endometriosis who have children 137.

The prognosis of early-stage OCCC is obviously superior to advanced OCCC. The 5-year overall survival (OS) and progression-free survival (PFS) rates are 80%~89% and 56%~88% for International Federation of Gynecology and Obstetrics (FIGO) stages I and II and decrease to 52% and 25% for stages III and IV, respectively^138^, ^139^. Among patients at early-stage, survival is better in OCCC compared to serous adenocarcinoma. However, OCCC patients at late stage tended to have poorer prognosis than those with other histological subtypes of EOC, especially in advanced EOC stages with 1.65-fold higher hazard rate for death 12, 140. Several studies also indicated patients with endometriosis-associated OCCC had a better prognosis than non-endometriosis associated group which may be explained endometriosis-associated OCCC was more likely to be first diagnosis at younger age, lower stages and poor aggressiveness. Kumar retrospectively compared 42 cases of EAOC with 184 cases of non-endometriosis associated ovarian carcinoma (non-EAOC) and reported that the median survival (199 vs 62 months) and the 5 year survival (62% vs 51%) of EAOC was better than non-EAOC 126. Association with endometriosis may be a crucial predictor of better survival in ovarian cancer.

## Prevention of endometriosis-associated OCCC

Clinicians should be aware of the increased risk of endometriosis-associated OCCC in high-risk populations and select appropriate preventive measures. Long-term use of oral contraceptives is a promising strategy to prevent ovarian cancer. Studies on the relationship between oral contraceptives and ovarian cancer indicate reduced ovarian cancer risk with oral contraceptive use 141. Two recent large studies have focused on the effects of tubal ligation in decreasing the risk of ovarian cancer 142. This risk reduction was the greatest for clear-cell and endometrioid carcinoma 143. Tubal ligation has a protective effect on these two subtypes of ovarian cancer, which is believed to be related to the prevention of retrograde menstruation, ovarian seeding by endometrial cells, and inflammation. Evidence suggests that oophorectomy reduces ovarian cancer risk effectively. Investigators have generally reported that unilateral oophorectomy and radical extirpation of all visible endometriosis following an endometriosis diagnosis have a good protective effect on the subsequent development of ovarian cancer 144. The ovarian cancer risk increases with the age at which ovarian endometriosis is diagnosed, and this increased risk is more significant in women aged ≥50 years 145. Based on these data, active surgical intervention should be considered for older women suffering from endometriosis.

## Conclusions

Patients with endometriosis have a higher risk of developing OCCC is compelling. OCCC is a specific pathological type of EOC with unique molecular and clinical features. We have reported a variety of molecular events such as ARID1A mutations, PIK3CA mutations, MET activation, HNF-1β activation, and miRNA dysfunction in endometriosis-associated OCCC. Besides, micro-environmental factors such as oxidative stress, immune cell dysfunction, inflammation, steroid hormones, and stem cells also play crucial roles in the carcinogenesis. Endometriosis-associated OCCC seems to be a special entity that differ from those of other histological types. OCCC is chemoresistant, has a poor prognosis, and is often difficult to treat. To improve the survival of OCCC patients, it is urgent to develop novel treatments based on its molecular alterations. The ARID1A, PI3K/AKT/mTOR, MET, and HNF-1β pathways are frequently activated and are considered promising targets for the treatment of endometriosis-associated OCCC. Some targeted inhibitors for these pathways are currently undergoing preclinical trials to evaluate whether they can be used as a treatment strategy for endometriosis-associated OCCC. The feasibility and efficiency of new target drugs in patients with endometriosis-associated OCCC should also be studied in future clinical trials.

## Figures and Tables

**Table 1:** Critical genetic changes in endometriosis-associated OCCC

Genes	Gene type	Change	Pathways affected	Roles in tumor development	References
ARID1A	Tumor suppressor	Mutation in 46-57%	SWI/SNF complex; PI3K/AKT/mTOR	Modulate accessibility of transcription factors to promoters	25, 56-70
PIK3CA	Oncogenic	Mutation in 33-43%	PI3K/AKT/mTOR	Proliferation/survival	36, 73-83
MET	Oncogenic	Amplification in 24-37%	Ras/Raf/MEK/ERK; PI3K/AKT/mTOR	Proliferation/survival	84-89
HNF-1β		Over 95% positive	HNF-1β	Stimulation of transcription	88, 91-97
XRCC5				DNA repair	52, 114
PTCH2			hedgehog signaling pathway	Proliferation/survival	52, 111
eEF1A2	Oncogenic		PI3K/AKT/mTOR	Apoptosis	52, 109, 110
miR-191			TIMP3	Proliferation/invasion	98, 102

**Table 2:** Clinical comparison of OCCC and ovarian high-grade serous carcinoma

OCCC	Ovarian high-grade serous carcinoma
Common in Asia	Common in Europe and America
More common in young patients	More common in elderly patients
More common in the early stage (I-II: 7-81%)	More common in the late stage (III-IV: 80%)
Associated with endometriosis	Associated with fallopian tube intraepithelial cancer
Resistant to first-line chemotherapy	Sensitive to first-line chemotherapy
ER/PR staining negative	ER/PR staining positive (65-96%)
HNF-1β (+) (over 95%)	HNF-1β (-)
High ARID1A mutations (46-57%)	No ARID1A mutations
High PIK3CA mutations (33-43%)	Rare PIK3CA mutations
